# High Prevalence of ESBL-Producing *Klebsiella pneumoniae* Causing Community-Onset Infections in China

**DOI:** 10.3389/fmicb.2016.01830

**Published:** 2016-11-15

**Authors:** Jing Zhang, Kai Zhou, Beiwen Zheng, Lina Zhao, Ping Shen, Jinru Ji, Zeqing Wei, Lanjuan Li, Jianying Zhou, Yonghong Xiao

**Affiliations:** ^1^Collaborative Innovation Center for Diagnosis and Treatment of Infectious Diseases, State Key Laboratory for Diagnosis and Treatment of Infectious Disease, The First Affiliated Hospital, School of Medicine, Zhejiang UniversityHangzhou, China; ^2^Department of Respiratory Disease, The First Affiliated Hospital, School of Medicine, Zhejiang UniversityHangzhou, China; ^3^Department of Clinical Laboratory, The First Affiliated Hospital of Soochow UniversitySoochow, China

**Keywords:** community-onset infections, CTX-M, resistance mechanisms, sequence type, hypervirulent *K. pneumoniae*

## Abstract

The aim of this work was to investigate the epidemiological and genetic characteristics of ESBL-producing *Klebsiella pneumoniae* (ESBL-Kp) causing community-onset infections. *K. pneumoniae* isolates were collected from 31 Chinese secondary hospitals between August 2010 and 2011. Genes encoding ESBL and AmpC beta-lactamases were detected by PCR. The isolates were assigned to sequence types (STs) using multi-locus sequence typing (MLST). Eleven ESBL-Kp strains were selected for whole-genome sequencing (WGS) for investigating the genetic environment and plasmids encoding ESBL genes. A total of 578 *K. pneumoniae* isolates were collected, and 184 (31.8%) carried ESBL genes. The prevalence of ESBL-Kp varied from different geographical areas of China (10.2–50.3%). The three most prevalent ESBL genes were *bla*_CTX-M-14_ (*n* = 74), *bla*_CTX-M-15_ (*n* = 60), and *bla*_CTX-M-3_ (*n* = 40). MLST assigned 127 CTX-M-14 and CTX-M-15 producers to 54 STs, and CC17 was the most prevalent population (12.6%). STs (23, 37, and 86) that were known frequently associated with hypervirulent *K. pneumoniae* (hvKP) account for 14.1% (18/127). Phylogenetic analysis by concatenating the seven loci of MLST revealed the existence of ESBL-producing *K. quasipneumoniae* (two strains) and *K. varricola* (one strain), which was further confirmed by WGS. This study highlights the challenge of community-onset infections caused by ESBL-Kp in China. The prevalence of STs frequently associating with hvKP should be of concern. Surveillance of ESBL-KP causing community-onset infections now appears imperative.

## Introduction

Extended-spectrum β-lactamases (ESBLs) have increased dramatically among clinical *Enterobacteriaceae* isolates during last three decades. In the last two decades, CTX-M replaces SHV as the major type of ESBLs disseminating worldwide (Zhao and Hu, 2013; Calbo and Garau, 2015). Surveillance in Asia, Latin America, and European revealed dramatically increasing resistance to cephalosporins amongst *Escherichia coli* and *Klebsiella* spp., largely contingent on the spread of CTX-M ESBLs (Kumarasamy et al., 2010; Zhao and Hu, 2013). In most of the Europe countries, CTX-M-15 is the most prevalent ESBL type (Pitout, 2013), and recently also disseminated in America, Canada, and Latin America (Denisuik et al., 2013; Wang G. et al., 2013; Kazmierczak et al., 2015). In China, *bla*_CTX-M-14_ is identified as the most prevalent ESBL gene (An et al., 2012; Yang et al., 2015).

It has been suggested that the frequent acquirement of plasmids harboring *bla*_CTX-M_ is largely responsible for the increase of CTX-M-producing *Enterobacteriaceae*. The *bla*_CTX-M_ genes are found to associate with certain replicon types of plasmids, mainly including IncF, IncI, IncN, IncHI2, IncL/M, and IncK groups (Zhao and Hu, 2013). The IncF group (FIA, FIB, and FII) is believed to play an important role in the dissemination of *bla*_CTX-M-15_ gene, and IncF, IncK and IncI1 largely contribute to the dissemination of *bla*_CTX-M-14_ gene. The *bla*_CTX-M-3_ gene is mainly harbored by plasmids of IncL/M and IncI1, and *bla*_CTX-M-9_ gene by IncHI2 plasmids (Zhao and Hu, 2013). Multiple mobile genetic elements, e.g., IS*Ecp1* and IS*CR1*, are involved in the mobilization of *bla*_CTX-M_ genes as well (Shahid et al., 2012; Zhao and Hu, 2013).

Extended-spectrum β-lactamase-producing *K. pneumoniae* (ESBL-Kp) has recently become an important nosocomial pathogen (Arpin et al., 2009; Maina et al., 2012; Pitout, 2013; Lohiya et al., 2015). In the tertiary hospitals of China, approximate 50% nosocomial-acquired infections are caused by ESBL-Kp and *bla*_CTX-M-15_ and *bla*_CTX-M-14_ are the predominant genotypes (Wang Q. et al., 2013; Li et al., 2014; Yang et al., 2015). Recently, the prevalence rate of ESBL-Kp in community-acquired infections is increasing even causing invasive infection (Kassakian and Mermel, 2014; Toubiana et al., 2016). However, limited knowledge about the dissemination of community-onset ESBL-Kp on national scale in China, especially in settings of secondary hospitals or primary health care. This study was to investigate the epidemiological and genetic characteristics of ESBL-Kp isolates causing community-onset infections in 31 secondary hospitals across China.

## Materials and Methods

### Collection of Clinical Isolates

Isolates were collected from August 2010 to 2011 in 31 secondary hospitals locating in 11 provinces representing seven major geographic regions of China (listed in **Supplementary Table S1**). Patients were selected for this study using the criteria as previous described (Zhang et al., 2014). Bacterial strains were isolated from clinical specimens (urine, blood, sputum, abscesses, and secretions) and identified using API20 (bioMérieux, Durham, NC, USA). All the pure cultures were frozen at -80°C and shipped to our laboratory for re-identification and further analysis as described previously (Zhang et al., 2014).

### Antimicrobial Susceptibility Testing

Minimum inhibitory concentrations (MICs) of 18 antimicrobial agents (ampicillin, piperacillin, cefazolin, cefuroxime, ceftazidime, ceftriaxone, cefepime, ampicillin-sulbactam, piperacillin-tazobactam, cefoperazone/sulbactam, cefoxitin, biapenem, imipenem, meropenem, amikacin, gentamicin, ciprofloxacin, levofloxacin, and fosfomycin) were determined by agar dilution method according to Clinical and Laboratory Standards Institute (CLSI, 2016). The breakpoint of biapenem was interpreted according to the recommended point of imipenem by CLSI (CLSI, 2016). The breakpoint interpretation of cefoperazone/sulbactam referred to that of cefoperazone and sulbactam. ESBL phenotype was confirmed by the standard double disc synergy test (DDST) with cefotaxime (30 ug) and ceftazidime (30 ug) alone and in combination with clavulanic acid (10 ug; Oxoid Limited, UK) as recommended by CLSI. *E. coli* ATCC 25922 and *K. pneumoniae* ATCC 700603 were used as quality control.

### Detection of Genes Encoding β-Lactamase and *K. pneumoniae*-Carbapenemase (KPC)

PCR amplification was used to detect β-lactamase genes (*bla*_CTX-M_, *bla*_SHV_, *bla*_TEM_, *bla*_OXA-1_ group, *bla*_OXA-10_ group, *bla*_V EB_, *bla*_PER_, *bla*_GES_, *bla*_CMY -1_ group, *bla*_CMY -2_ group, and *bla*_DHA_) and KPC gene (*bla*_KPC_). Primers used for PCR detection and sequencing were acquired from a previous study (Zhang et al., 2014). Sequencing results were analyzed online using Basic Local Alignment Search Tool (BLAST), and were further refined with use of a β-lactamase database^1^.

### Multi-Locus Sequence Typing (MLST)

Multi-Locus Sequence Typing was performed on 127 ESBL-Kp (69 *bla*_CTX-M-14_, 58 *bla*_CTX-M-15_) using the scheme of Institute Pasteur as described previously (Diancourt et al., 2005). New alleles and STs were assigned by the MLST database^2^. Clonal analysis of MLST data was performed using eBURST v3 (Feil et al., 2004). Clonal complexes (CCs) were defined as groups of two or more independent isolates that shared identical alleles at six loci. Each complex was named after the putative founder ST. A minimal spanning tree (MST) was generated by using BioNumerics v7.0 (Applied Maths, Sint-Martens-Latem, Belgium) to provide a graphical representation of the clonal distribution of ESBL-Kp. Neighbor-joining trees were constructed from concatenated sequences of seven MLST loci using the MEGA6 program with Kimura’s two-parameter model (Tamura et al., 2011).

### Whole-Genome Sequencing (WGS) and Data Analysis

Eleven ESBL-Kp were selected to WGS for further analysis of genetic environment of ESBL genes and plasmid characteristics, including one CTX-M-3-producing isolate, two CTX-M-9-producing isolates, seven CTX-M-14-producing isolates, and one CTX-M-15-producing isolate. Genomic DNA was extracted using QIAamp DNA Mini Kit (Qiagen, Hilden, Germany) and sequenced via HiSeq 2000 (Illumina, San Diego, CA, USA) with a 2 × 125 bp paired-end strategy. *De novo* assembly was generated by using the CLC Genomics Workbench, version 8.0.3 (CLC bio, Aarhus, Denmark). The plasmid analysis was performed as described previously (Zhou et al., 2015). In brief, the plasmids backbones were derived dependent on BLASTn. The contigs of each sample were blasted against the reference plasmid and plotted by BLAST Ring Image Generator (BRIG) (Alikhan et al., 2011).

### Accession Numbers

All sequence data of six novel SHV variants are assigned in the GenBank database under the following accession numbers: KC688280, KC688281, KC688282, KC688283, KC688284, and KC688285.

This Whole Genome Shotgun project has been deposited at GenBank under the accession LYWN00000000, LYWO00000000, LYWP00000000, LYWQ00000000, LYWR00000000, LYWS00000000, LYWT00000000, LYWU00000000, LYWV00000000, LYWW00000000, LYWX00000000. The version described in this paper is version XXXX01000000.

## Results

### Specimen Types and Patient Demographics

A total of 578 *K. pneumoniae* isolates were collected and identified from sputum (73.9%), urine (11.2%), blood (5.5%), abscess (3.3%), throat swab (2.4%), and unknown specimen (3.6%). In total, 61.2% of isolates were obtained from males. The distribution of patients’ age groups was as follows: ≤1 years, 20.2%; 2–17 years, 5.6%; 18–45 years, 20.2%; 46–64 years, 22.2%; and ≥65 years, 31.7%.

### Prevalence of ESBL-Kp Largely Varied across the Surveyed Regions

The results of antimicrobial susceptibility tests are summarized in **Table 1**. All isolates were susceptible to biapenem, and only one isolate was resistant to imipenem and meropenem. Additionally, 92.4, 91.5, and 91.4% of isolates were susceptible to amikacin, cefoperazone-sulbactam and fosfomycin, respectively. Notably, 184 *K. pneumoniae* isolates (31.9%) showed ESBL phenotype with resistance to cefuroxime (97.9%), cefazolin (97.3%), ceftriaxone (96.8%), piperacillin (96.8%), gentamicin (68.1%), and ampicillin-sulbactam (67.9%). The prevalence of ESBL-Kp varied from 10.2 to 50.3% across different regions (**Table 2**).

**Table 1:** Results for susceptibility tests and Minimum inhibitory concentrations (MICs) for *K. pneumoniae* strains (*n* = 587) isolated from 31 county hospitals.

	All isolates (*n* = 578)					ESBL-positive (phenotype) isolates (*n* = 184)				
		
Antibiotic	Resistant %	Susceptible %	MIC50(mg/L)	MIC90(mg/L)	MIC range	Resistant %	Susceptible %	MIC50(mg/L)	MIC90(mg/L)	MIC range
Ampicillin	86.3	2.3	64	>256	0.5–1024	99	0.5	>256	>256	1–1024
Piperacillin	38.1	56.7	8	>256	0.03–1024	96.8	0	>256	>256	32–1024
Ampicillin-sulbactam	26.1	60.9	8	64	0.03–1024	67.9	10	32	128	0.125–1024
Cefoperazone-sulbactam	2.5	91.5	1	16	0.03–512	7.3	74.3	8	32	0.125–512
Piperacillin-tazobactam	1.3	88.2	4	32	0.015–1024	3.7	70	16	32	2–1024
Cefazolin	39.4	59.9	2	256	0.5–256	97.3	2.7	256	256	0.5–256
Cefuroxime	37	59.9	4	256	0.125–256	97.9	1.1	256	256	4–256
Ceftazidime	14.7	81.2	0.25	32	0.015–256	44	45	8	128	0.015–256
Ceftriaxone	31.2	68.1	0.125	64	0.03–512	96.8	1.6	32	128	1–512
Cefepime	11.5	83.3	0.5	32	0.03–1024	33.5	51.3	8	128	0.03–1024
Cefoxitin	10.2	86.4	4	32	0.25–512	30.4	65.4	4	128	1–512
Biapenem	0	100	0.064	0.125	0.015–1	0	100	0.064	0.125	0.015–1
Imipenem	0.2	99.8	0.25	0.25	0.015–16	0.5	99.5	0.25	0.25	0.015–16
Meropenem	0.2	99.8	0.032	0.125	0.015–16	0.5	99.5	0.016	0.125	0.015–16
Amikacin	7.6	92.4	2	8	0.03–512	20.5	79.5	2	128	0.06–512
Gentamicin	30.1	68.6	1	128	0.125–512	68.1	30.9	64	128	0.25–512
Ciprofloxacin	15	80.6	0.125	16	0.015–256	31.4	57.1	1	64	0.015–256
Levofloxacin	11.6	86.5	0.125	8	0.006–256	23.7	71.6	1	16	.015–256
Fosfomycin	4.7	91.4	16	64	0.25–256	8.1	85.9	16	128	0.5–256


**Table 2:** Geographical distribution of ESBL-producing *K. pneumoniae* isolates in seven regions of China.

ESBL genotype	No. of isolates (prevalence, %)							
	
	North (*n* = 71)	Northwest (*n* = 171)	Northeast (*n* = 72)	East (*n* = 49)	South (*n* = 52)	Central (*n* = 20)	Southwest (*n* = 143)	Total (*n* = 578)
CTX-M-1 group	8	33	12	1	10	2	10	76
CTX-M-15	2	22	9	1	1	1	4	40
CTX-M-55	3	1			2	1	1	8
CTX-M-3	3	9	3		7		5	27
CTX-M-101		1						1
CTX-M-9 group	6	25	6	4	5	4	6	56
CTX-M-14	6	23	5	4	5	4	5	52
CTX-M-9		2						2
CTX-M-27							1	1
CTX-M-65			1					1
SHV-type	5	5	1		3	1	2	17
SHV-27	3	2			3		1	9
SHV-2		2					1	3
SHV-12	2	1	1					4
SHV-41						1		1
CTX-M-1+9 groups		15	3			2	1	21
CTX-M-14 +CTX-M-3		6	1			2		9
CTX-M-14+ CTX-M-15		6	2					8
CTX-M-15+ CTX-M-24							1	1
CTX-M-15+ CTX-M-9		3						3
CTX-M+SHV	3	9	1		1			14
CTX-M-3+SHV-2	2		1					3
CTX-M-3+SHV-12	1							1
CTX-M-15+SHV-12					1			1
CTX-M-14+SHV-2		1						1
CTX-M-14+SHV-27		1						1
CTX-M-15+SHV-27		1						1
CTX-M-14+CTX-M-15+ SHV-27		2						2
CTX-M-14+CTX-M-15+ SHV-38		1						1
CTX-M-15+CTX-M-9+ SHV-27		3						3

Total	22 (31.0%)	86 (50.3%)	23 (31.9%)	5 (10.2%)	20 (38.5%)	9 (45.0%)	19 (13.3%)	184 (31.8%)


### CTX-M-14 and CTX-M-15 Were the Predominant ESBL Genotypes

The *bla*_CTX-M_ genes were detected in 90.8% (*n* = 167) of the 184 ESBL-Kp isolates. CTX-M-1 group and CTX-M-9 group genes were identified in 109 and 85 isolates, respectively, and both gene groups co-existed in 27 isolates (**Table 2**). The *bla*_CTX-M-14_ gene was the most prevalent one (*n* = 74), followed by *bla*_CTX-M-15_ (*n* = 60), *bla*_CTX-M-3_ (*n* = 40), *bla*_CTX-M-55_ (*n* = 8), *bla*_CTX-M-9_ (*n* = 8), and *bla*_CTX-M-27_, *bla*_CTX-M-24_, *bla*_CTX-M-65_, and *bla*_CTX-M-101_ (*n* = 1 for each).

Various SHV genes were detected in 31 ESBL isolates (19.1%), including SHV-27 (*n* = 16), SHV-2 (*n* = 7), SHV-12 (*n* = 6), SHV-41 (*n* = 1), and SHV-38 (*n* = 1). Additionally, nine isolates carried an inhibitor-resistant SHV variant SHV-26. The concomitance of SHV and CTX-M genes was identified in 14 isolates. VEB-1 was detected in one isolate, co-existed with *bla*_CTX-M-9_ and *bla*_CTX-M-15_.

Two AmpC genes CMY-2 and DHA-1 were detected in three and 32 isolates, respectively. All the 35 isolates carrying AmpC genes were also ESBL producers. Additionally, KPC-2 gene was detected in one strain.

### Clones Frequently Associated with Hypervirulent *K. pneumoniae* Nationally Disseminated

**FIGURE 1:**
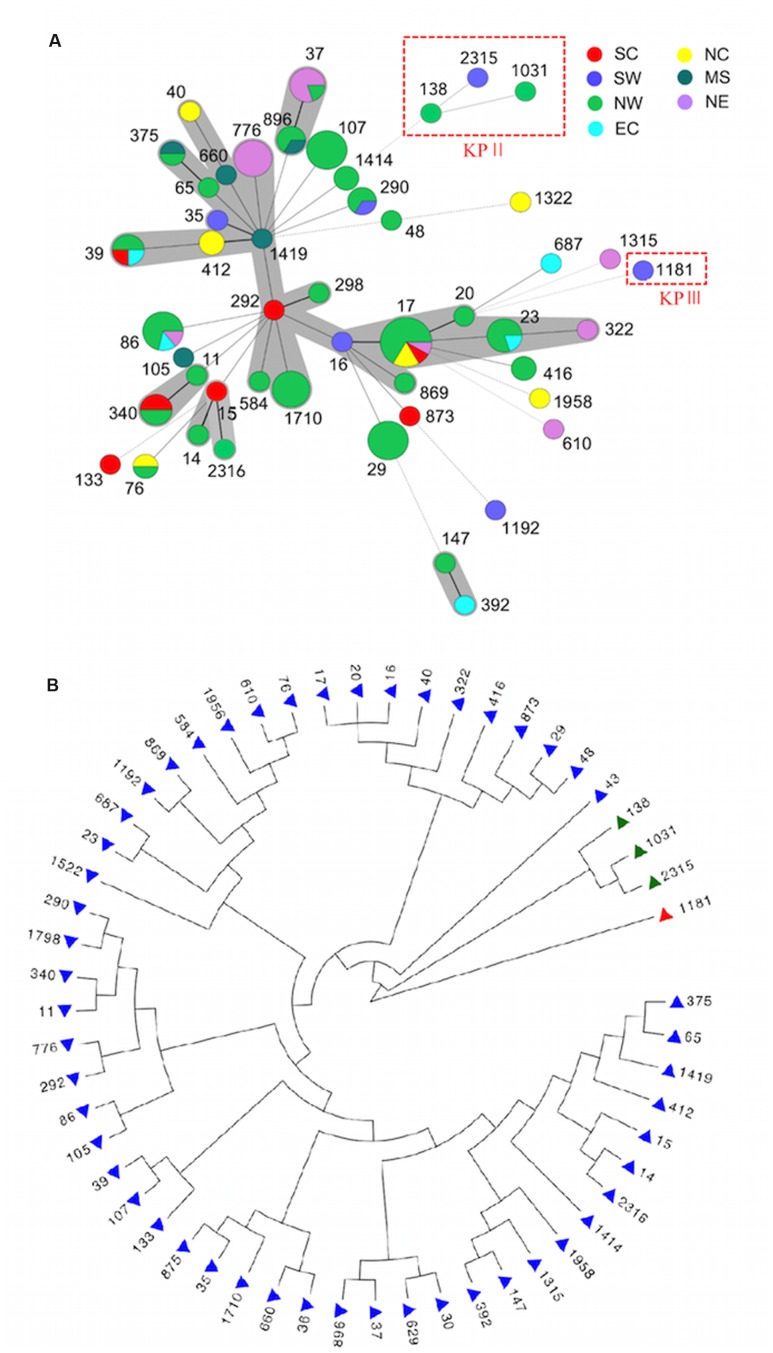
**Genetic relationship of ESBL-KP.** **(A)** Minimum-spanning tree (MST) illustrating STs in relation to districts. STs belonging to KpII and KpIII were marked in the red rectangle, and the remaining STs belong to KPI. Thick lines represent single-locus variants, dashed lines correspond to double-locus variants and dotted lines describe three to seven allele differences between the sequence types. **(B)** Phylogenetic tree of 127 ESBL-KP as determined on the basis of the allelic profiles of the seven MLST genes. Branch in blue indicate phylogroup KpI, green indicate KpII (*K. quasipneumoniae*) and red indicate KpIII (*K. variicola*). NC, Northern China; NW, North western China; NE, North eastern China; EC, Eastern China; SC, Southern China; MS, Central southern China; SW, South western China.

Multi-locus sequence typing analysis showed an extensive diversity in 127 ESBL-Kp that 54 STs were identified and no predominant ones existed. The minimum-spanning tree (MST) showed no correlation between STs and geographic distributions (**Figure 1A**). Six CCs comprising 45 isolates were identified by eBURST (**Supplementary Figure S1**), and the remaining 82 isolates belong to 37 singletons. CC17 (including ST16, ST17, and ST20) was the most prevalent population accounting for 12.6% (16/127), followed by ST86 (*n* = 7), ST107 (*n* = 7), ST873 (*n* = 7), ST37 (*n* = 6), ST776 (*n* = 6), ST23 (*n* = 5), ST290 (*n* = 5). Of note, ST23, ST37, and ST86 account for 14.1% (18/127), and these STs are known frequently associating with hypervirulent *K. pneumoniae* (hvKP) (Jung et al., 2013; Bialek-Davenet et al., 2014; Cubero et al., 2016). The major carriers of CTX-M-15 were ST86 (*n* = 7), ST873 (*n* = 6), and ST776 (*n* = 6), and those of CTX-M-14 included ST17 (*n* = 12) and ST107 (*n* = 7).

### ESBL-Producing *K. varricola* and *K. quasipneumoniae* were Identified by Phylogenetic Analysis

Phylogenetic analysis by concatenating sequences of seven MLST loci showed that 54 STs detected in this study were split into three distinct clades, and most STs belong to clade I (**Figure 1B**). It is known that *K. pneumoniae* consisted of three phylogenetic groups (KPI, II, and III), and has recently been reclassified as three different species (*K. pneumoniae. K. quasipneumoniae*, and *K. variicola*) (Brisse and Verhoef, 2001; Rosenblueth et al., 2004; Brisse et al., 2014). Nine isolates of clade I (ST17, ST290, ST322, ST875, ST896, ST1031, ST1522, and ST1798) and two isolates of clade II (ST138 and ST1031) were consequently sent to WGS for the species determination. Phylogenomic analysis assigned clade I to *K. pneumoniae* (KPI), and clade II to *K. quasipneumoniae* (KPII) (**Figure 1B**). The clade III was then supposed to be *K. varricola*. The two *K. quasipneumoniae* isolates carried *bla*_CTX-M-9_ (ST138) and *bla*_CTX-M-14_ (ST1031), respectively, and the one *K. varricola* strain carried *bla*_CTX-M-15_ (ST1181).

### The Transmission Mechanism of CTX-M Genes

**FIGURE 2:**
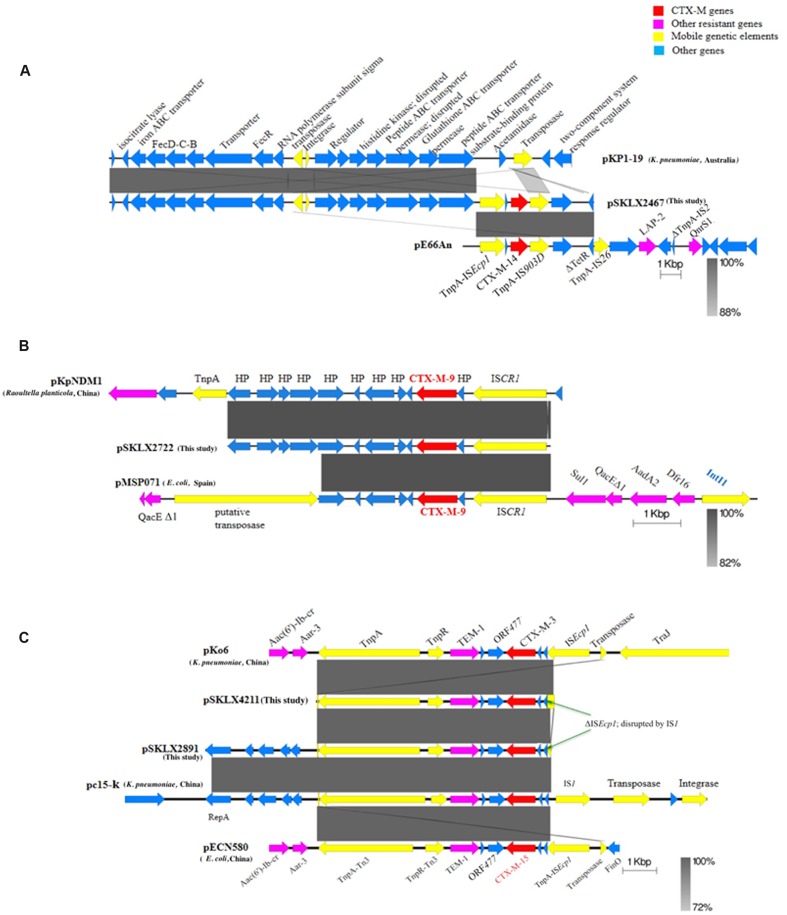
**Schematic representation of the *bla*_CTX-M_ environment.** Arrows represent ORFs and their direction of transcription. **(A)** Genetic environment of *bla*_CTX-M-14_ and homologous sequences in plasmid pKP1-9 and pE66An to illustrate the relationship of the three plasmids. **(B)** Genetic environment of *bla*_CTX-M-9_ and homologous sequences in plasmid pKPNDM1 and pMSP071 to illustrate the relationship of the three plasmids. **(C)** Genetic environment of *bla*_CTX-M-3_ and *bla*_CTX-M-15_. pSKLX4211 and pKo6 encoded *bla*_CTX-M-3_ and pSLKX2891, pc15-k and pECN580 were CTX-M-15 carriers. The *bla*_CTX-M-3_ and *bla*_CTX-M-15_ shared an identical genetic environment.

Plasmids and the genetic environment were analyzed to determine the transmission mechanism of CTX-M genes. The seven CTX-M-14-producing isolates (including *K. pneumoniae* ST17, ST1798, ST896, ST290, ST322, and *K. quasipneumoniae* ST1031) collected from different geographical regions shared a highly homologous plasmid carrying *bla*_CTX-M-14_ gene, of which the backbone was highly similar with pKP1-19 (accession number CP0012884) identified in a *K. pneumoniae* strain isolated in the environment in Australia (**Supplementary Figure S2A**). Of note, pKP1-19 did not encode any drug resistance genes. The seven strains shared an identical genetic environment structure as IS*Ecp1-bla*_CTX-M-14_-IS*903* (**Figure 2A**).

Both CTX-M-9 isolates (including *K. quasipneumoniae* ST138 strain and *K. pneumoniae* ST2316) harbored a similar CTX-M-9 plasmid showing a highly similar backbone with plasmid pFB2.1 carried by *Pluralibacter gergoviae* isolated in Malaysia (accession number CP014776) (**Supplementary Figure S2B**). Blasting the contigs harboring *bla*_CTX-M-9_ in GenBank revealed that the best match was pKPNDM1 (accession number JX515588; 99% coverage and 100% identity) carried by a *Raoultella planticola* strain isolated from a patient with post surgical operation infection. This was the only best match showed no less than 99% coverage of the contig. Notably, the *R. planticola* strain was isolated from the northwest of China (Gansu province), where the two *K. pneumoniae* were isolated. An IS*CR1* located upstream of the *bla*_CTX-M-9_ gene (**Figure 2B**).

The CTX-M-3 isolate harbored a plasmid carrying the CTX-M-3 gene, of which the backbone was highly similar to a *K. pneumoniae* plasmid pKF3-94 (accession number FJ876826) with CTX-M-15 gene detected from Zhejiang province of China (**Supplementary Figure S2C**). Blasting the 7050-bp contig carrying *bla*_CTX-M-3_ gene in GenBank revealed some best matches with 99% coverage and 99% identity, including pHS08204 (accession number KP125893; CTX-M-15 plasmid of *K. pneumoniae* HS08204, from Shanghai), pECN580 (accession number KF914891; CTX-M-15 plasmid of *E. coli*, from Beijing), and pKo6 (accession number KC958437; CTX-M-3 plasmid of *K. pneumoniae*, from Shanghai), pKOX_R1 (accession number CP003684; CTX-M-3 plasmid of *K. oxytoca*, from Taiwan), pc15-k (accession number HQ202266; CTX-M-15 plasmid of *K. pneumoniae*, from Guangzhou), and pKF3-94. These plasmids were detected from different regions of China with different backbones. Examining the genome of the CTX-M-15-producing strain identified a CTX-M-15 plasmid sharing a highly homologous backbone with a *K. pneumoniae* plasmid pc15-k (**Supplementary Figure S2D**). Blasting the 10043-bp contig harboring the *bla*_CTX-M-15_ gene revealed the same batch of best matches as those of CTX-M-3-producing plasmids mentioned above (**Figure 2C**).

The *bla*_CTX-M-3_ and *bla*_CTX-M-15_ shared an identical genetic environment. A truncated IS*Ecp1* disrupted by IS*1* (at the end of the contig) was found at the upstream of *bla*_CTX-M-3/15_ genes, and genes encoding TEM-1, TnpR of Tn3, and TnpA of Tn3 located downstream of *bla*_CTX-M-3/15_ gene (**Figure 2C**).

## Discussion

This study illustrated the epidemiological and genetic characteristics of ESBL-Kp causing community-onset infections in 31 secondary hospitals distributed in areas across China. Our results showed that the prevalence rate of community-onset ESBL-Kp (31.8%) was comparable to that of nosocomial-acquired ESBL-Kp revealed by multiple studies across China (30.1–39.7%) (An et al., 2012; Yang et al., 2013). This raises the concern that the dissemination of ESBL-Kp in community has become another challenge for the resistance control in China, which would be even more difficult to be controlled than that in health-care systems.

As the spread pattern of nosocomial-acquired ESBL-Kp, polyclonal dissemination without any predominant clones was identified in this community-onset study. However, the community-onset ESBL-Kp showed a different clonal distribution comparing to that of nosocomial-acquired ESBL-Kp in China. A multi-center study on tertiary hospitals in China showed that ST11 was the most prevalent ST (12.2%) among 74 STs identified in 155 ESBL-Kp (An et al., 2012), whereas ST17 was the most prevalent one in our study (12.6%). This is concordant with a previous study that ST17 was predominant in CTX-M-14-Kp and CTX-M-15-Kp more likely correlating to community-onset infections (Peirano et al., 2012). CTX-M-14 and CTX-M-15 were the predominant ESBL genotypes in our community-onset collections, which is the same as that found in the nosocomial studies. However, the population structure of CTX-M-14 and CTX-M-15 producers was different between our collections and the nosocomial-acquired isolates. A multi-center study identified that ST37, 5, 505, 11, and 23 were the major carriers of CTX-M-14, and CTX-M-15 mainly associated with two epidemic clones ST340 and ST15 (An et al., 2012). In our study, the major carriers of CTX-M-15 were ST86 (*n* = 7), ST873 (*n* = 6), and ST776 (*n* = 6), and the major CTX-M-14 producers consisted of ST17 (*n* = 12) and ST107 (*n* = 7). Neither CTX-M-15 nor CTX-M-14 associate with any known epidemic clones implies that different clones of community-onset ESBL-Kp would become epidemic in the future.

Of note, we found a set of ESBL-producing clones (ST23, ST37, and ST86) known to frequently associate with hvKP. Especially, ST86 was the most abundant clone among CTX-M-15 producers in this study, and is known to correlate to invasive infections such as bacteraemia, pneumonia with septic shock and liver abscess (Bialek-Davenet et al., 2014; Liao et al., 2014; Passet and Brisse, 2015). In this study, five of seven ST86 strains were isolated from sputum of children (aged from 15 days to 3 years) with community-acquired respiratory tract infections, and the other two were isolated from liver abscess and blood in adult patients. To our best knowledge, this is the first report of community dissemination of ST86 in Mainland China. Further study is needed to determine whether the ST86 strains are hvKP and why they emerged in respiratory tract infections in children.

Further investigations revealed various transmission mechanisms employed by different CTX-M genes in the community dissemination. We found that plasmids carried CTX-M-14 identified in seven phylogenetically diverse isolates collected from distinct geographical areas across China shared a similar backbone (**Supplementary Figure S2**). This suggests that a batch of plasmids sharing a similar backbone could be an important reservoir for *bla*_CTX-M-14_ in community in China. Interestingly, the segment carrying *bla*_CTX-M-9_ identified in our two isolates got a unique best match (99% coverage and 100% identity) to a part of *R. planticola* plasmid pKPNDM1. Moreover, the *R. planticola* strain was isolated from the same region (Gansu province) with the two isolates. This suggests that *bla*_CTX-M-9_ might not via the horizontal transfer of CTX-M-9-IS*CR1* but that of the whole segment as found in the study spread in that region. Additionally, we found that an identical genetic environment (*Δ*IS*Ecp1-bla*_CTX-M_*-orf477-bla*_TEM-1_-*tnpR-tnpA*) shared by *bla*_CTX-M-15_ and *bla*_CTX-M-3_ identified in two phylogenetically diverse isolates collected from distinct geographical areas. This indicated that both *bla*_CTX-M-15_ and *bla*_CTX-M-3_ might spread mainly via the horizontal transfer of this structure in the community in China.

*K. pneumoniae* was previously classified into three phylogenetic groups (KPI, II, and III), which now are assigned to three different species (*K. pneumoniae. K. quasipneumoniae*, and *K. variicola*), respectively. *K. quasipneumoniae* (KPII) and *K. variicola* (KPIII) were previously thought to be more susceptible than *K. pneumoniae* (KpI) isolates to antimicrobial agents, and less frequently cause infections (Brisse et al., 2004; de Melo et al., 2011; Holt et al., 2015). *K. variicola* is an environmental bacterium mainly associated with plants. Rare reports are available of infections caused by *K. quasipneumoniae* and *K. variicola* (Holt et al., 2015). Maatallah et al. (2014) found that *K. variicola* infections correlate to higher mortality comparing to *K. pneumoniae* infections. Few studies showed the ESBL-producing KPII and KPIII strains can cause nosocomial infections (Valverde et al., 2008; Maatallah et al., 2014; Holt et al., 2015). The present study revealed the emergency of ESBL-producing *K. quasipneumoniae* and *K. variicola* causing community-onset infections. The lack of accurate identification methods for the three species may underestimate the severity of *K. quasipneumoniae* and *K. variicola* infections. Surveillance of ESBL-producing *K. quasipneumoniae* and *K. variicola* would be helpful to prevent the further prevalence.

In summary, this study highlights the challenge of ESBL dissemination in community in China. Various transmission mechanisms are responsible for the spread of the most common CTX-M genes in community. The prevalence of STs frequently associating with hvKP should be of concern. Surveillance of ESBL-KP causing community-onset infections now appears imperative.

## Author Contributions

Conceived and designed the experiments: JZ, YX, KZ, LZ, BZ, LL, and JZ. Performed the experiments: JZ, JJ, ZW, PS, and LZ. Analyzed the data: JZ, KZ, and YX. Wrote the paper: JZ and KZ.

## Conflict of Interest Statement

The authors declare that the research was conducted in the absence of any commercial or financial relationships that could be construed as a potential conflict of interest.

## Footnotes

1 http://www.lahey.org/Studies/

2 http://bigsdb.web.pasteur.fr/
