# Supplementary material for: High Prevalence of ESBL-Producing Klebsiella pneumoniae Causing Community-Onset Infections in China
Source: Front Microbiol. 2016 Nov 15;7:1830. doi: 10.3389/fmicb.2016.01830 (PMC5109008; doi:10.3389/fmicb.2016.01830)
Supplement: Supplementary file 1 [file Data_Sheet_1.docx]

***Supplementary Material***

# High prevalence of ESBL-producing *Klebsiella pneumoniae* causing community-onset infections in China

**Jing Zhang^1, 2#^, Kai Zhou^1#^, Beiwen Zheng^1^, Lina Zhao^3^, Ping Shen^1^, Jinru Ji^1^, Zeqing Wei^1,^** ^†^**, Lanjuan Li^1^, Jianying Zhou^2^* and Yonghong Xiao^1^***

*** Correspondence:** Corresponding Author: zjyhz@zju.edu.cn or xiao-yonghong@163.com

## 1. Supplementary Figures and Tables

For more information on Supplementary Material and for details on the different file types accepted, please see [here](http://home.frontiersin.org/about/author-guidelines#SupplementaryMaterial).

## 2.1 Supplementary Figures

**
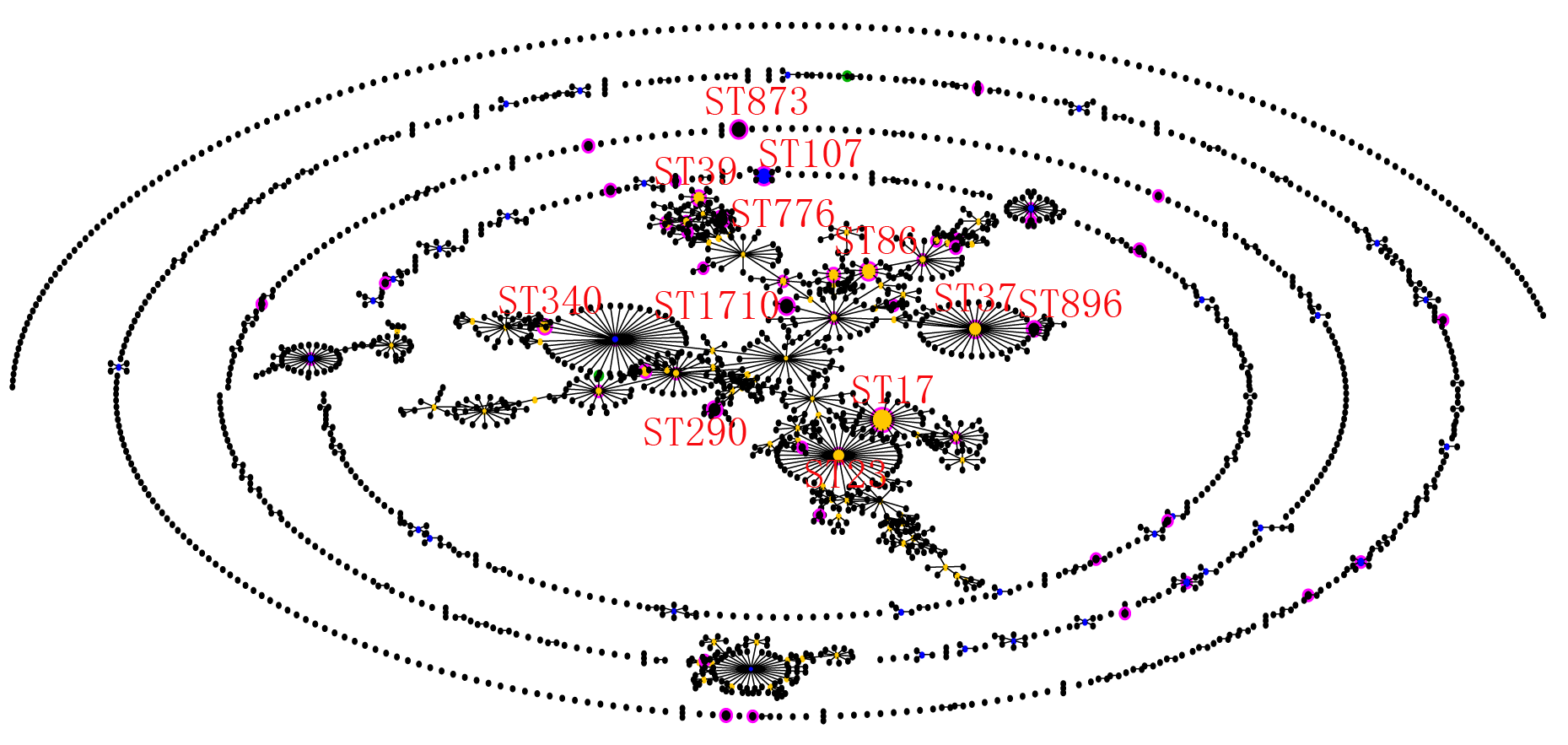
**

**Supplementary Figure S1.** eBurst analysis of allelic profiles of 127 ESBL-Kp. STs including a minimum of 4 isolates and/or identified in this study are indicated by numbers; those identified in the present study are additionally labeled by purple. Subgroup founders are shown in yellow. The relative size of the circles indicates the prevalence of STs and lines between STs connect single locus variants (SLVs).


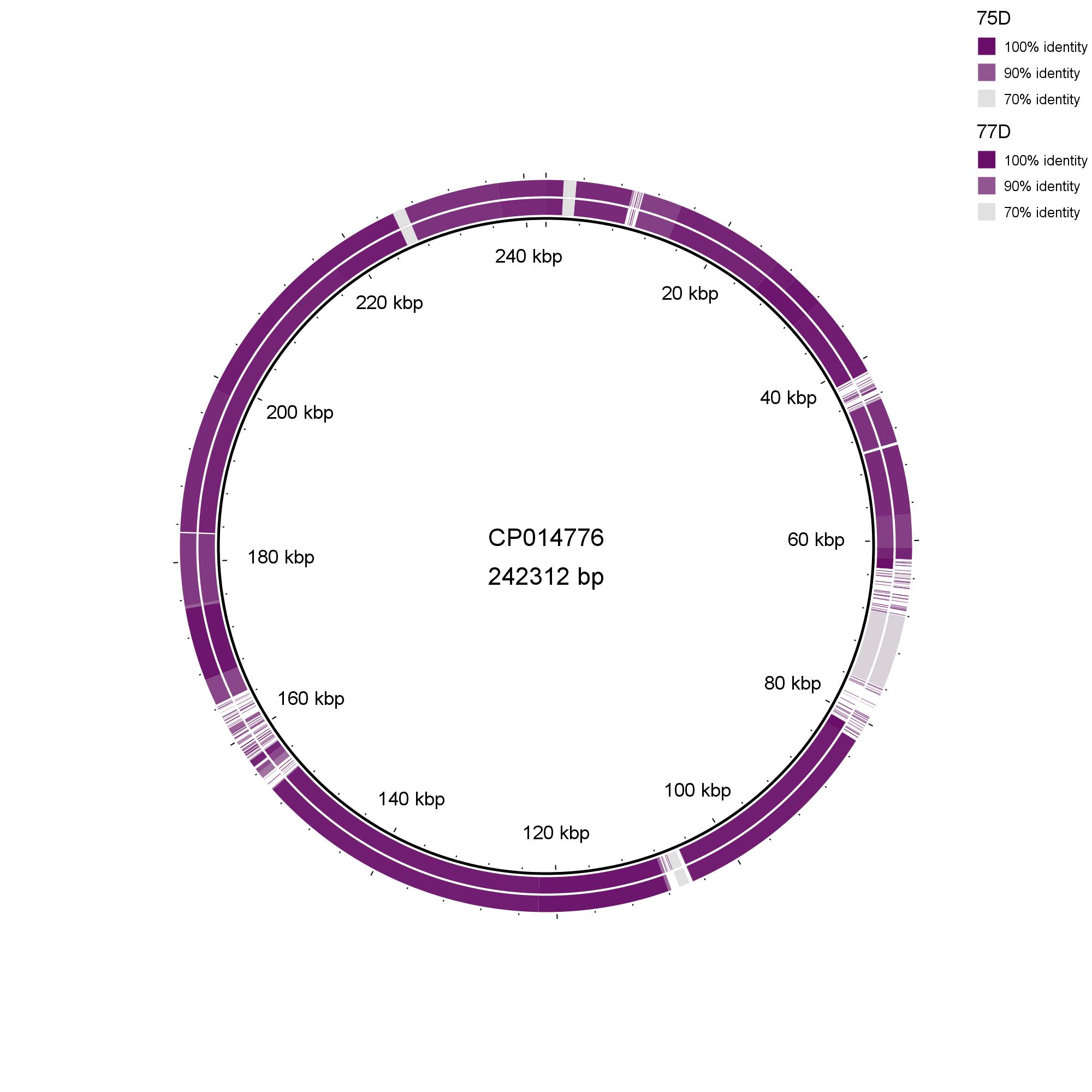


a)


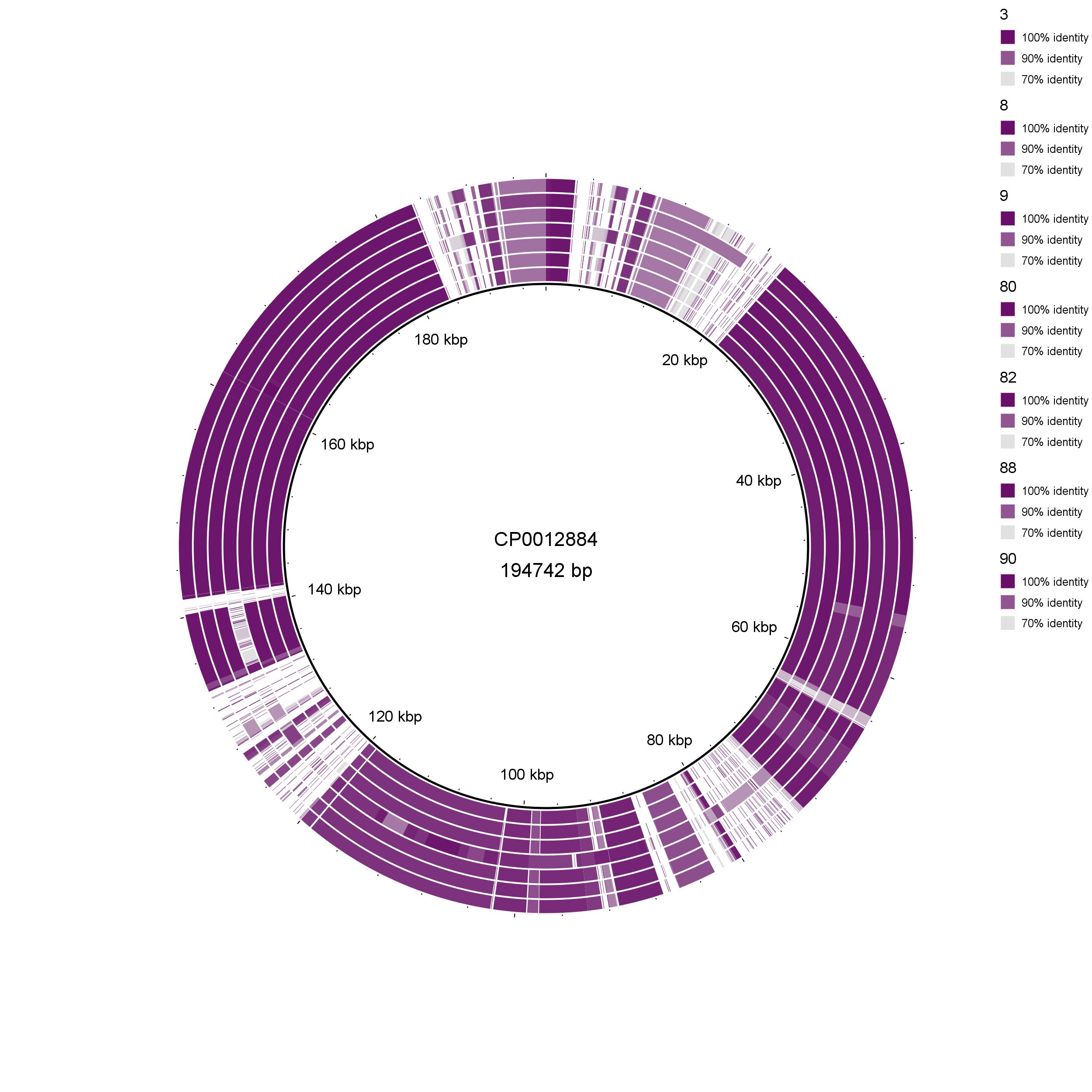


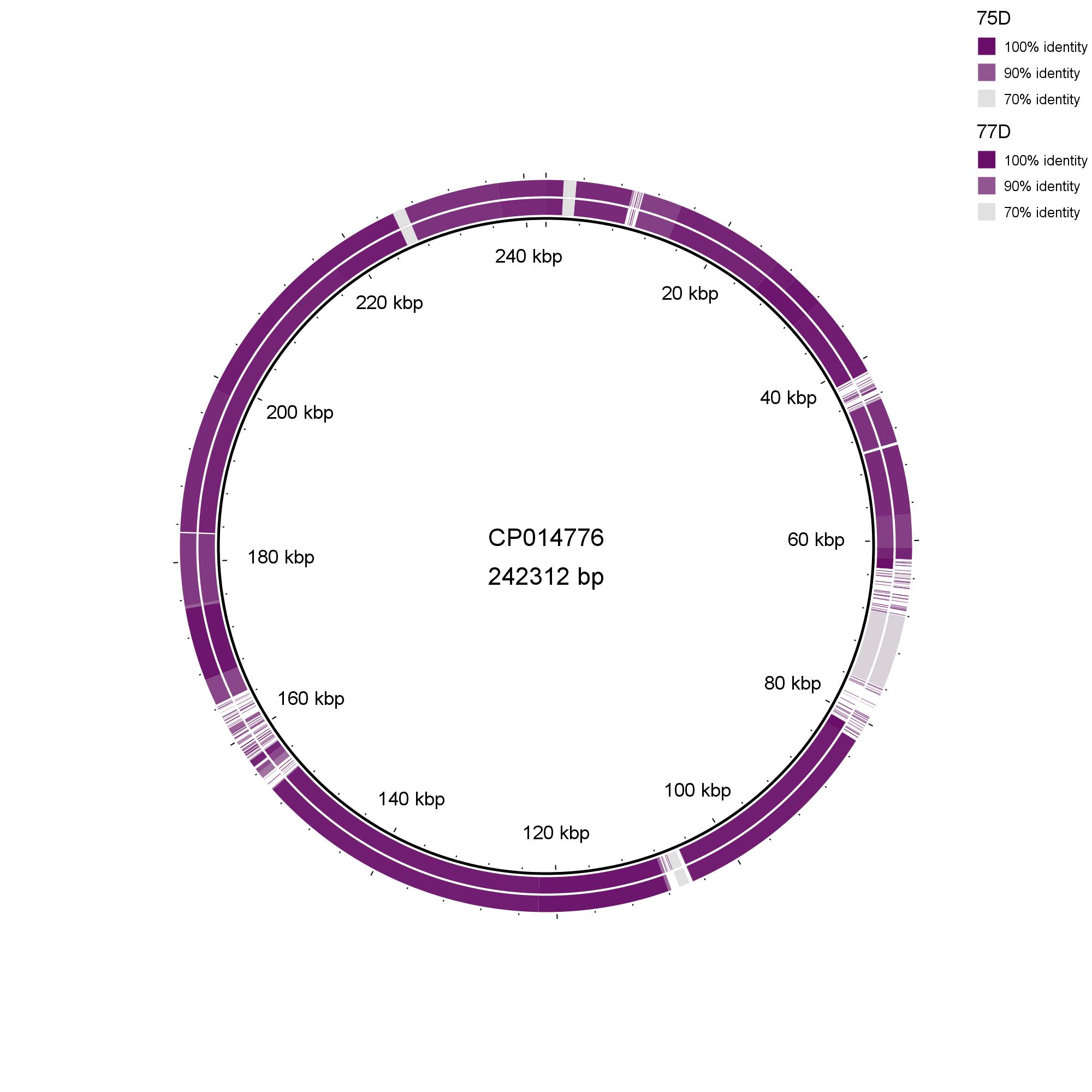


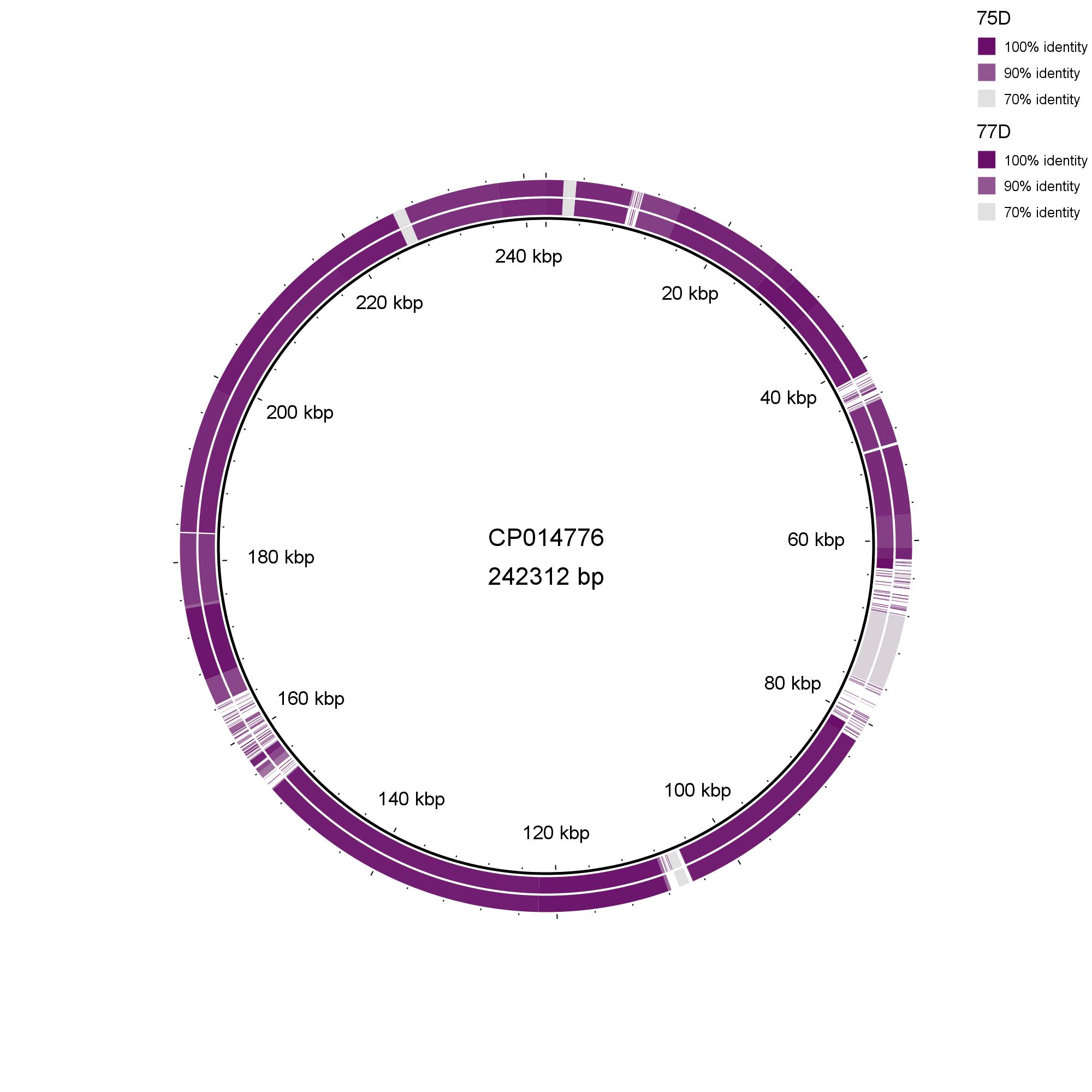


b)


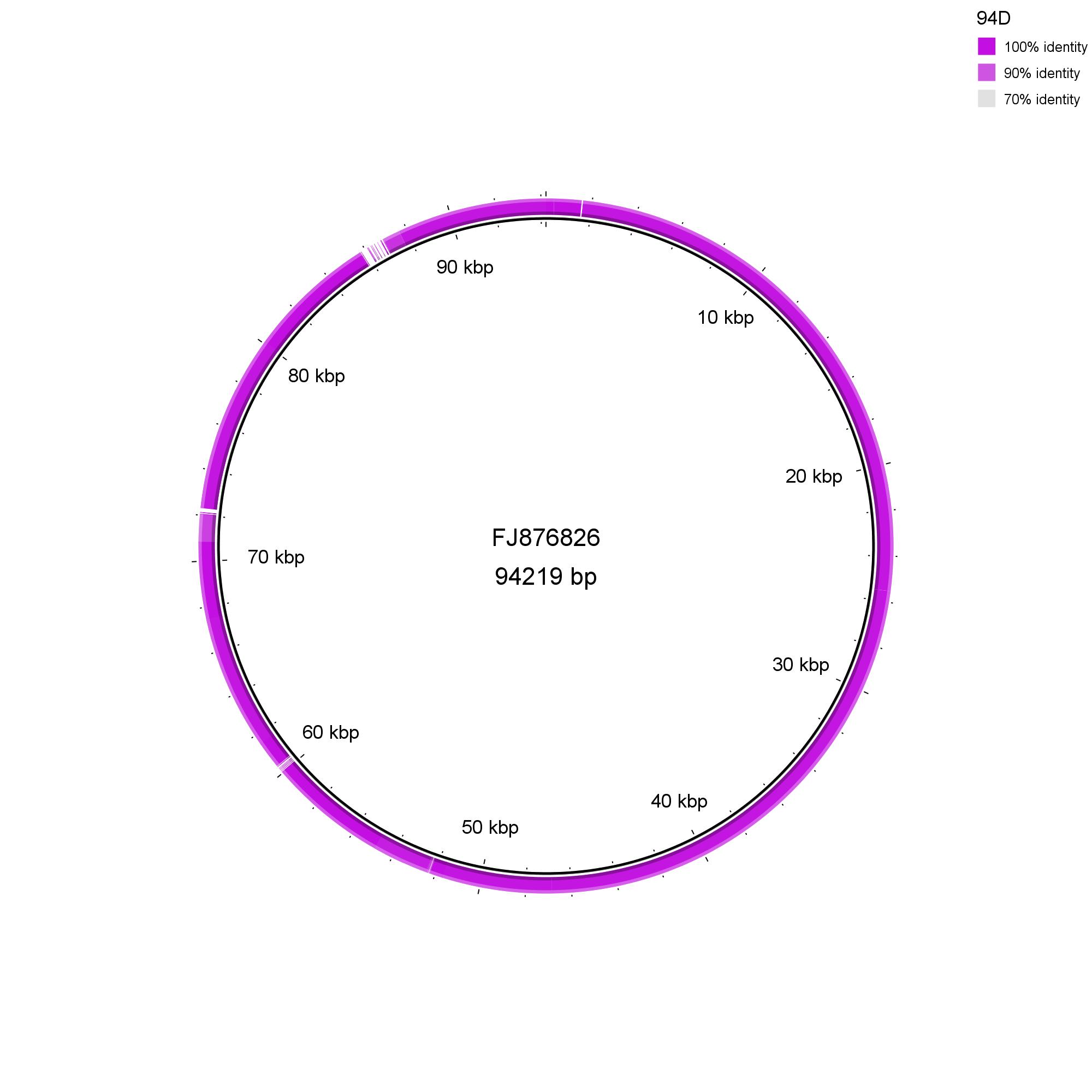


c)


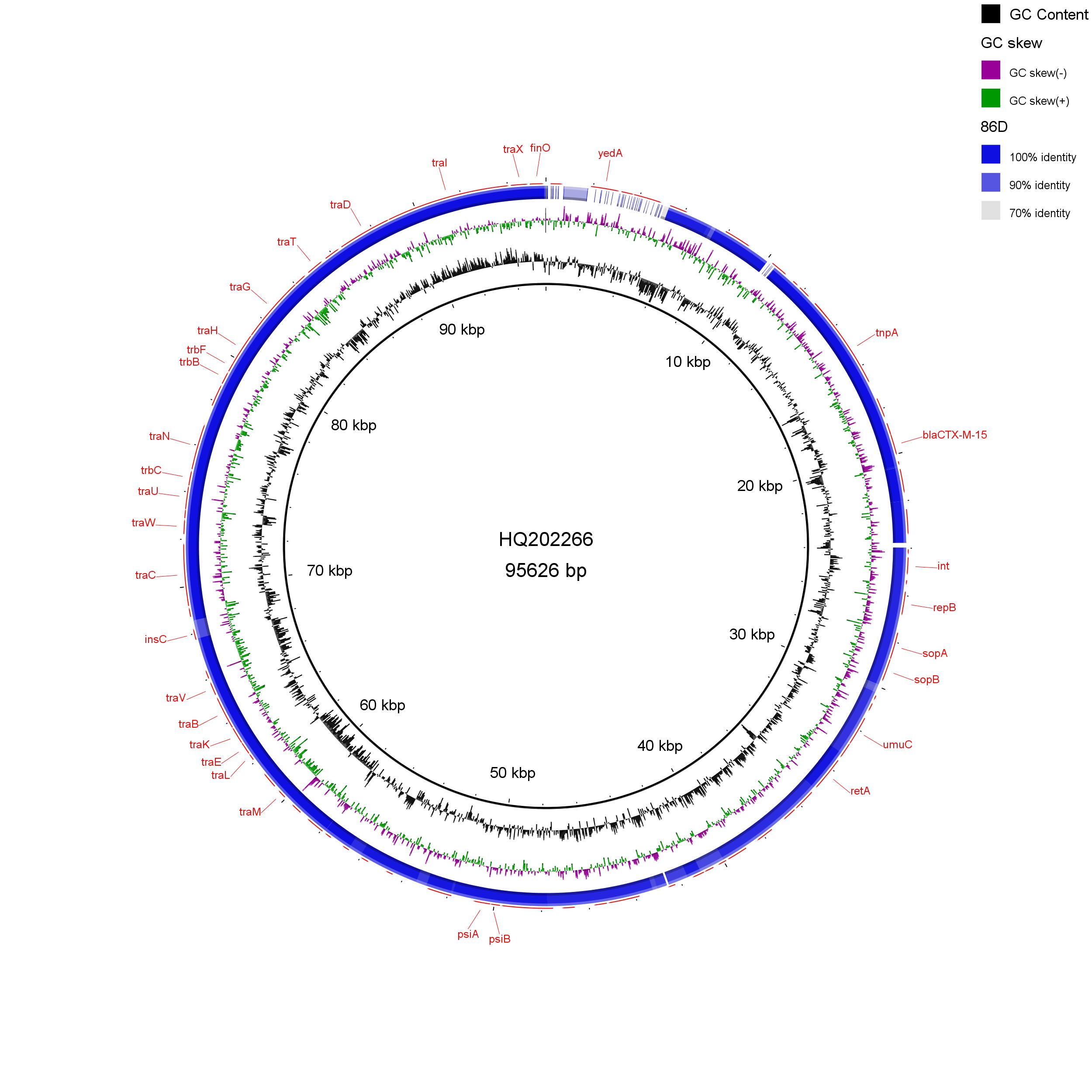


d)

**Supplementary Figure S2.** Comparison of the backbone of plasmid encoded *bla*_CTX-M_ with previous plasmid: a) backbone of seven plasmids encoded *bla*_CTX-M-14_ with pKP1-19 (from *K. pneumoniae* strain isolated in the environment in Australia); b) both CTX-M-9 plasmids showing a highly similar backbone with plasmid pFB2.1 carried by *Pluralibacter gergoviae* isolated in Malaysia; c) plasmid carrying the CTX-M-3 gene showing was highly similar backbone with a plasmid pKF3-94 encoded *bla*_CTX-M-15_ detected from *K. pneumoniae* Zhejiang province of China; d) backbone of two plasmids encoded *bla*_CTX-M-15_ with pc15-k (CTX-M-15 plasmid of *K. pneumoniae,* from Guangzhou of China)

**2.2 Supplementary Table**

**Supplementary Table S1: List of 31 participant county hospitals**

| Region* | Hospital name | Serial number |
| --- | --- | --- |
| NC | Second People's Hospital of Hengshui | NC1 |
| NC | Qinhuangdao military hospital | NC2 |
| NC | Linhe district People's Hospital of Inner Mongolia | NC3 |
| NC | Zalantun People's Hospital Inner Mongolia | NC4 |
| NC | Jishan County People's Hospital of Shanxi Province | NC5 |
| NC | Tianjin Beichen Hospital | NC6 |
| NW | First People's Hospital in Urumqi, Xinjiang | NW1 |
| NW | Hetian People's Hospital | NW2 |
| NW | Ili Kazak Autonomous Prefecture Chinese Medicine Hospital | NW3 |
| NW | Second Division hospital of Xinjiang Korla | NW4 |
| EC | Taixing People's Hospital | EC1 |
| EC | Shangyu People's Hospital | EC2 |
| EC | Shaowu Municiple Hospital of Fujian Province | EC3 |
| SC | Shenzhen Baoan People's Hospital | SC1 |
| SC | Longgang Central Hospital | SC2 |
| SC | Panyu District People's Hospital of Guangzhou | SC3 |
| SC | Foshan Shunde Longjiang Hospital | SC4 |
| SC | Dongguan Hengli Hospital | SC5 |
| MS | Second People's Hospital of Jingzhou | MS1 |
| MS | Yiling Hospital of Yichang | MS2 |
| MS | Zhijiang People's Hospital of Hubei | MS3 |
| MS | Yingshan People's Hospital of Hubei | MS4 |
| MS | Jianli County People's Hospital of Hubei | MS5 |
| NE | Jinzhou Hospital of Dalian | NE1 |
| NE | Shenyang Women and Children's Hospital | NE3 |
| NE | Yingkou Development Zone Hospital | NE4 |
| SW | Shifang People's Hospital of Sichuan | SW1 |
| SW | Jiangyou People's Hospital of Sichuan | SW2 |
| SW | Mianyang People's Hospital of Sichuan | SW3 |
| SW | Renshou People's Hospital of Sichuan | SW4 |
| SW | Suining First People's Hospital of Sichuan | SW5 |

*Abbreviation: NC, Northern China (Tianjin, Inner Mongolia, Hebei, and Shanxi province); North western China (Xinjiang); North eastern China (Liaoning); EC, Eastern China (Fujian and Zhejiang province); SC, Southern China (Guangzhou province); MS, Central Southern China (Hubei province); SW, South western China (Sichuan province).
